# Spatiotemporal dynamics and influencing factors of human brucellosis in Mainland China from 2005–2021

**DOI:** 10.1186/s12879-023-08858-w

**Published:** 2024-01-11

**Authors:** Meng Zhang, Xinrui Chen, Qingqing Bu, Bo Tan, Tong Yang, Liyuan Qing, Yunna Wang, Dan Deng

**Affiliations:** Department of Epidemiology and Health Statistics, Chongqing Medical University of Public Health, Chongqing, 500000 China

**Keywords:** Brucellosis, MGWR, Spatial epidemiology, Medical meteorology, Prevention, Control policy

## Abstract

**Background:**

Brucellosis poses a significant public health concern. This study explores the spatial and temporal dynamic evolution of human brucellosis in China and analyses the spatial heterogeneity of the influencing factors related to the incidence of human brucellosis at the provincial level.

**Methods:**

The Join-point model, centre of gravity migration model and spatial autocorrelation analysis were employed to evaluate potential changes in the spatial and temporal distribution of human brucellosis in mainland China from 2005 to 2021. Ordinary Least Squares (OLS), Geographically Weighted Regression (GWR), and Multi-scale Geographically Weighted Regression (MGWR) models were constructed to analyze the spatial and temporal correlation between the incidence rate of human brucellosis and meteorological and social factors.

**Results:**

From 2005 to 2021, human brucellosis in China showed a consistent upward trend. The incidence rate rose more rapidly in South, Central, and Southwest China, leading to a shift in the center of gravity from the North to the Southwest, as illustrated in the migration trajectory diagram. Strong spatial aggregation was observed. The MGWR model outperformed others. Spatio-temporal plots indicated that lower mean annual temperatures and increased beef, mutton, and milk production significantly correlated with higher brucellosis incidence. Cities like Guangxi and Guangdong were more affected by low temperatures, while Xinjiang and Tibet were influenced more by beef and milk production. Inner Mongolia and Heilongjiang were more affected by mutton production. Importantly, an increase in regional GDP and health expenditure exerted a notable protective effect against human brucellosis incidence.

**Conclusions:**

Human brucellosis remains a pervasive challenge. Meteorological and social factors significantly influence its incidence in a spatiotemporally specific manner. Tailored prevention strategies should be region-specific, providing valuable insights for effective brucellosis control measures.

## Background

Brucellosis is a zoonotic disease caused by the invasion of Brucella spp. into the body. Brucella can cause abortion and infertility in infected animals, and fever and systemic disease in humans. Humans are generally susceptible to brucellosis, and the main routes of infection are the respiratory tract, digestive tracts and broken mucocutaneous [1]. Brucellosis is one of the most widespread and dangerous zoonotic diseases in the world today. The brucellosis epidemic exists in over 170 countries and regions, causing billions of dollars in economic losses worldwide [2, 3]. In 1905, brucellosis first occurred in Shanghai, China. Subsequently, from the 1980s to the early 1990s, mainland China gradually initiated prevention and control activities for brucellosis [4]. The disease has become a major global public health problem as it not only threatens human well-being, reduces livestock productivity, and increases the medical burden but also poses a food safety risk.

Growing evidence indicates that brucellosis exhibits strong temporal and spatial characteristics [5–7]. With the launch of active surveillance for brucellosis in 2005, the prevention and control of brucellosis in all regions of China have been deepening under the unified guidance of the State, but there are differences in the history of development, natural environment, economic level, and dietary characteristics of the various regions, so that the spatial and temporal evolution of brucellosis, as well as the heterogeneity of morbidity factors at the interprovincial level, need to be further explored.However, existing studies often focus on independent temporal or spatial regression analyses, which have limitations. The time trend of brucellosis is assessed using traditional regression analysis, which primarily reflects the overall trend of global data and may not reveal specific trends in local data [8].In spatial analysis, linear regression or logistic regression models are commonly employed, assuming consistent regression coefficients across the entire study area and overlooking spatial non-stationarity. Consequently, the research outcomes may not fully capture the true spatial characteristics and spatial heterogeneity of influencing factors [9].

The Join-point model is an ideal approach for analyzing significant changes, turning points, and the direction and speed of sequence data. Utilizing this model allows for assessing disease change characteristics within different intervals of the global time range, providing a more scientific approach than intuitive descriptions of brucellosis trends based solely on common data tables and statistical graphs [10].In geographical distribution, the 'center of gravity' is a common method for studying the transfer of substances and energy. The center of gravity migration model is a crucial analytical tool for examining spatial changes in elements during the regional development process. As regional development involves continually changing the position of the center of gravity of elements, the movement of the element's center of gravity objectively reflects the evolutionary process [11]. Simultaneously, spatial autocorrelation analysis is conducted to assess the spatial correlation of brucellosis and the degree of correlation in geographical distribution.

With the advancement of geographic information technology, various spatial statistical analysis methods based on spatial econometrics have emerged. In geography, the closer the distance, the stronger the correlation (Tobler's first law), and if it is inappropriate to divide the area directly for traditional linear regression analyses, it is necessary to take into account the samples within a certain spatial range around each sample. Geographically Weighted Regression (GWR), introduced by scholars to study spatial relationships and correlations, can effectively detect spatial non-smoothness [12]. It has found wide applications in multidisciplinary fields [13–15]. However, GWR assumes the same optimal bandwidth for all explanatory variables, potentially leading to biased parameter estimates. All explanatory variables in a classical GWR model can only produce a uniform scale of action, but realistic factors affecting brucellosis have different scales of action at the spatio-temporal level. In 2017, Fotheringham et al. proposed a Multiscale Geographically Weighted Regression (MGWR) model, an extension of GWR that considers differential spatial bandwidths for independent variables. MGWR has become an effective tool for revealing the influence and scope of environmental factors.

In this study, we employed the joinpoint model, gravity migration model, and spatial autocorrelation to analyze the spatial and temporal evolution of brucellosis. Additionally, the MGWR model was used to explore spatial heterogeneity in factors influencing brucellosis incidence at the provincial level, with a view to providing scientific reference for the formulation of corresponding brucellosis prevention and control measures.

## Methods

### Data source

Data on the incidence of human brucellosis in China by region from 2005 to 2021 were obtained from the National Notifiable Infectious Disease Reporting Information System at the Chinese Center for Disease Control and Prevention (https://www.chinacdc.cn). Data on meteorological factors (such as average annual temperature, humidity, precipitation, etc.) and social factors (including the number of cattle stock, sheep stock, beef production, dairy production, total output value of the pastoral industry, regional GDP, etc.) were sourced from the National Bureau of Statistics of China (http://www.stats.gov.cn). Geographical information was acquired from the National Basic Geographic Information Centre (http://www.ngcc.cn/), and the maps of China referenced in this study are based on the standard map with review number GS (2022) 4309, which was downloaded from the standard map service website of the Ministry of Natural Resources (http://bzdt.ch.mnr.gov.cn/).

### Statistical analysis

#### Temporal trends

Considering the issues of data redundancy and comparability, mainland China was divided into seven regions based on different dimensions such as development history, natural environment, economic level, dietary characteristics and ethnicity, and the methodology was developed by the state. In accordance with the seven natural geographic regions of China, the temporal characteristics of human brucellosis prevalence in each region were regressed in segments by building Join-point models. This method allows for analyzing turning points, determining their locations, specifying the direction of change, the turning points are selected by grid search method (GSM), which divides the space where the parameters are located into a grid, and each intersection point corresponds to a planning scheme, and then the performance index of the corresponding scheme is calculated point by point with a fixed step in a set interval to determine the optimal parameters. For the analysis of the Joinpoint model, the GSM creates a grid of all the possible locations of the "turning points", calculates the sum of squared errors (SSE) and mean squared errors (MSE) for each case, and selects the grid with the smallest MSE as the optimal turning point. The grid with the smallest MSE is selected as the optimal turning point, and calculating the annual percent change (APC) and average annual percent change (AAPC) to clarify the direction and speed of change for the whole and local areas, respectively. If the APC is greater than 0, the incidence rate is increasing from year to year, and vice versa. The formulas for APC and AAPC are as follows:

1 $$\mathrm{APC}=\left\{\mathrm{exp}(\upbeta )-\left.1\right\}\times 100\right.$$

2 $$\mathrm{AAPC}=\left\{\left.\mathrm{exp}(\frac{\sum {\mathrm{w}}_{\mathrm{i}}{\upbeta }_{\mathrm{i}}}{\sum {\mathrm{w}}_{\mathrm{i}}})-1\right\}\times 100\right.$$

where $\upbeta$ is the regression coefficient, ${\mathrm{w}}_{\mathrm{i}}$ is the number of years included in each segment, and ${\upbeta }_{\mathrm{i}}$ is the regression coefficient for each segment.

#### Spatial distribution

Centre of gravity calculation. Assuming that a certain study area consists of M sub-level areas i, where (${\mathrm{X}}_{\mathrm{i}}$, ${\mathrm{Y}}_{\mathrm{i}}$) denotes the latitude and longitude values of the ith sub-level area, and Mi represents the values of each factor in the sub-level area, the centre of gravity of each study factor is calculated by using the centre of gravity coordinate formula, calculated as for:

3 $$\mathrm{\rm X}=\frac{{\sum }_{\mathrm{i}=1}^{\mathrm{n}}{\mathrm{M}}_{\mathrm{i}}{\mathrm{X}}_{\mathrm{i}}}{{\sum }_{\mathrm{i}=1}^{\mathrm{n}}{\mathrm{M}}_{\mathrm{i}}}$$

4 $$\mathrm{Y}=\frac{{\sum }_{\mathrm{i}=1}^{\mathrm{n}}{\mathrm{M}}_{\mathrm{i}}{\mathrm{Y}}_{\mathrm{i}}}{{\sum }_{\mathrm{i}=1}^{\mathrm{n}}{\mathrm{M}}_{\mathrm{i}}}$$

The distance of inter-annual movement of the center of gravity spatial location, denoted as the Center of Gravity spatial shift distance, represents the shift in the center of gravity coordinates of a study factor from year j to year j + 1. This movement is expressed as the distance (D) between the center of gravity coordinates in different years, denoted by (${\mathrm{X}}_{\mathrm{j}+1}$,${\mathrm{Y}}_{\mathrm{j}+1}$) and (${\mathrm{X}}_{\mathrm{j}}$,${\mathrm{Y}}_{\mathrm{j}}$), where R is a constant set as 111.11 km., We used the following formula:

5 $${\mathrm{D}}_{(\mathrm{j}+1)-\mathrm{j}}=\mathrm{R}\times \sqrt{{({\mathrm{Y}}_{\mathrm{j}+1}-{\mathrm{Y}}_{\mathrm{j}})}^{2}+{({\mathrm{X}}_{\mathrm{j}+1}-{\mathrm{X}}_{\mathrm{j}})}^{2}}$$

The use of spatial econometric methods relies on the assumption of spatial heterogeneity among sample data. Therefore, a spatial autocorrelation analysis of the independent variables is necessary before constructing the GWR and MGWR models. Typically, a global spatial autocorrelation analysis is performed using Moran's *I* to determine whether the attribute values at the sample points under study exhibit spatial correlation with the values of the same attribute at other sample points within the domain. A negative index suggests discrete attribute values among the samples, while an index of 0 indicates that the attribute values are randomly distributed without significant spatial characteristics.The absolute value of I indicates the strength of correlation; a larger absolute value signifies stronger correlation, which is written in mathematical notation:

6 $$\mathrm{I}=\frac{{\sum }_{\mathrm{i}=1}^{\mathrm{n}}\sum_{\mathrm{j}=1}^{\mathrm{n}}({\mathrm{x}}_{\mathrm{i}}-\overline{\mathrm{x} })({\mathrm{x}}_{\mathrm{j}}-\overline{\mathrm{x} })}{{\mathrm{S}}^{2}\sum_{\mathrm{i}=1}^{\mathrm{n}}\sum_{\mathrm{j}=1}^{\mathrm{n}}{\mathrm{W}}_{\mathrm{ij}}}$$

I is Moran's *I* value, n refers to the number of spatial elements, ${\mathrm{x}}_{\mathrm{i}}$ and ${\mathrm{x}}_{\mathrm{j}}$ are the observed values at cell i and j, $\overline{\mathrm{x} }$ is the mean value of attribute values of all spatial units, while ${\mathrm{W}}_{\mathrm{ij}}$ refers to the spatial weight between elements i and j.

#### Model construction

To explore the factors affecting the incidence of brucellosis at the interprovincial level, we used ordinary least squares (OLS) as a starting point for spatial regression analyses; introduced spatial relationships using the GWR model, which is a fundamental method in spatial econometric statistics; and further constructed the MGWR model in conjunction with the study objectives, with the R^2^ and adjusted R^2^ measuring how well the regression models fit the data. Another key diagnostic, the modified Akaike's information criterion (AICc), was employed for model evaluation. Comparisons were made based on the R^2^, adjusted R^2^, and AICc values, where larger R^2^ and adjusted R^2^ values and smaller AICc values indicated better model fit.

The OLS model is a conventional linear regression model that only estimates the parameters in an average or global sense, but does not capture the spatial non-stationarity of the parameters. The model is generally expressed as.

7 $${\mathrm{Y}}_{\mathrm{i}}={\upbeta }_{0}+{\sum }_{\mathrm{k}}{\upbeta }_{\mathrm{k}}{\mathrm{X}}_{\mathrm{ik}}+{\upvarepsilon }_{\mathrm{i}}$$

where ${\mathrm{Y}}_{\mathrm{i}}$ denotes the dependent variable at the ith sample point, ${\upbeta }_{0}$ denotes the intercept of the linear regression equation, ${\upbeta }_{\mathrm{k}}$ denotes the regression coefficient of the kth independent variable, ${\mathrm{X}}_{\mathrm{ik}}$ denotes the kth independent variable at the ith sample point, and ${\upvarepsilon }_{\mathrm{i}}$ denotes the random error.

The GWR model is an improved model based on the traditional linear regression model. Its main advantage is the ability to apply the spatial weight matrix to the linear regression model, which will better demonstrate the spatial structural variation of the results. The model can be expressed as.

8 $${\mathrm{Y}}_{\mathrm{i}}={\upbeta }_{0}({\mathrm{u}}_{\mathrm{i}},{\mathrm{v}}_{\mathrm{i}})+{\sum }_{\mathrm{k}}{\upbeta }_{\mathrm{k}}{({\mathrm{u}}_{\mathrm{i}},{\mathrm{v}}_{\mathrm{i}})\mathrm{X}}_{\mathrm{ik}}+{\upvarepsilon }_{\mathrm{i}}$$

where ${\mathrm{Y}}_{\mathrm{i}}$ is the dependent variable; ${\mathrm{X}}_{\mathrm{ik}}$ denotes the kth independent variable at the i-th sample point, ${\upbeta }_{\mathrm{k}}({\mathrm{u}}_{\mathrm{i}},{\mathrm{v}}_{\mathrm{i}})$ is the regression coefficient of the kth independent variable at $({\mathrm{u}}_{\mathrm{i}},{\mathrm{v}}_{\mathrm{i}})$; and ${\upvarepsilon }_{\mathrm{i}}$ denotes the random error. These parameters are obtained using weighted least squares estimation.

The MGWR model is an extended model based on the GWR model assigned to the differential bandwidth of different independent variables to describe the differential scale of action of different independent variables, which can make full use of the spatial heterogeneity of the sample data and improve the accuracy of parameter estimation. The model is presented as follows.

9 $${\mathrm{Y}}_{\mathrm{i}}={\upbeta }_{0}({\mathrm{u}}_{\mathrm{i}},{\mathrm{v}}_{\mathrm{i}})+\sum\nolimits_{\mathrm{i}=1}^{\mathrm{m}}{\upbeta }_{\mathrm{bw}\cdot \mathrm{k}}{({\mathrm{u}}_{\mathrm{i}},{\mathrm{v}}_{\mathrm{i}})\mathrm{X}}_{\mathrm{ik}}+{\upvarepsilon }_{\mathrm{i}}$$

In this case, ${\upbeta }_{\mathrm{bw}\cdot \mathrm{k}}$ represents the differential bandwidth of the independent variable, and the other symbols have the same meaning as in Eq. (2). MGWR first presets the parameters as those of the GWR model when estimating the model parameters, and then uses the back-fitting algorithm to correct the model parameters.

Based on the fitted coefficients of the MGWR model, the spatiotemporal distribution of the variables was plotted. The natural break-point method was used to group the data with high similarity. When the fit coefficient is positive, it indicates that the independent variable has a facilitating effect on the dependent variable; conversely, it indicates that the independent variable has a suppressing effect on the dependent variable, and the larger the absolute value of the fit coefficient, the greater the degree of effect. Temporal trend analysis was carried out using Join-point Regression Software (4.9.1.0) software, and mapping, model construction and model parameter estimation were carried out using ArcGIS (10.8) software, MGWR (2.2) software. Developed by professional teams, these software tools ensure robustness and credibility. Results were considered significant at *P* < 0.05 for readability and acceptability.

## Results

### Temporal trends in the national brucellosis epidemic from 2005 to 2021

**Fig. 1:**
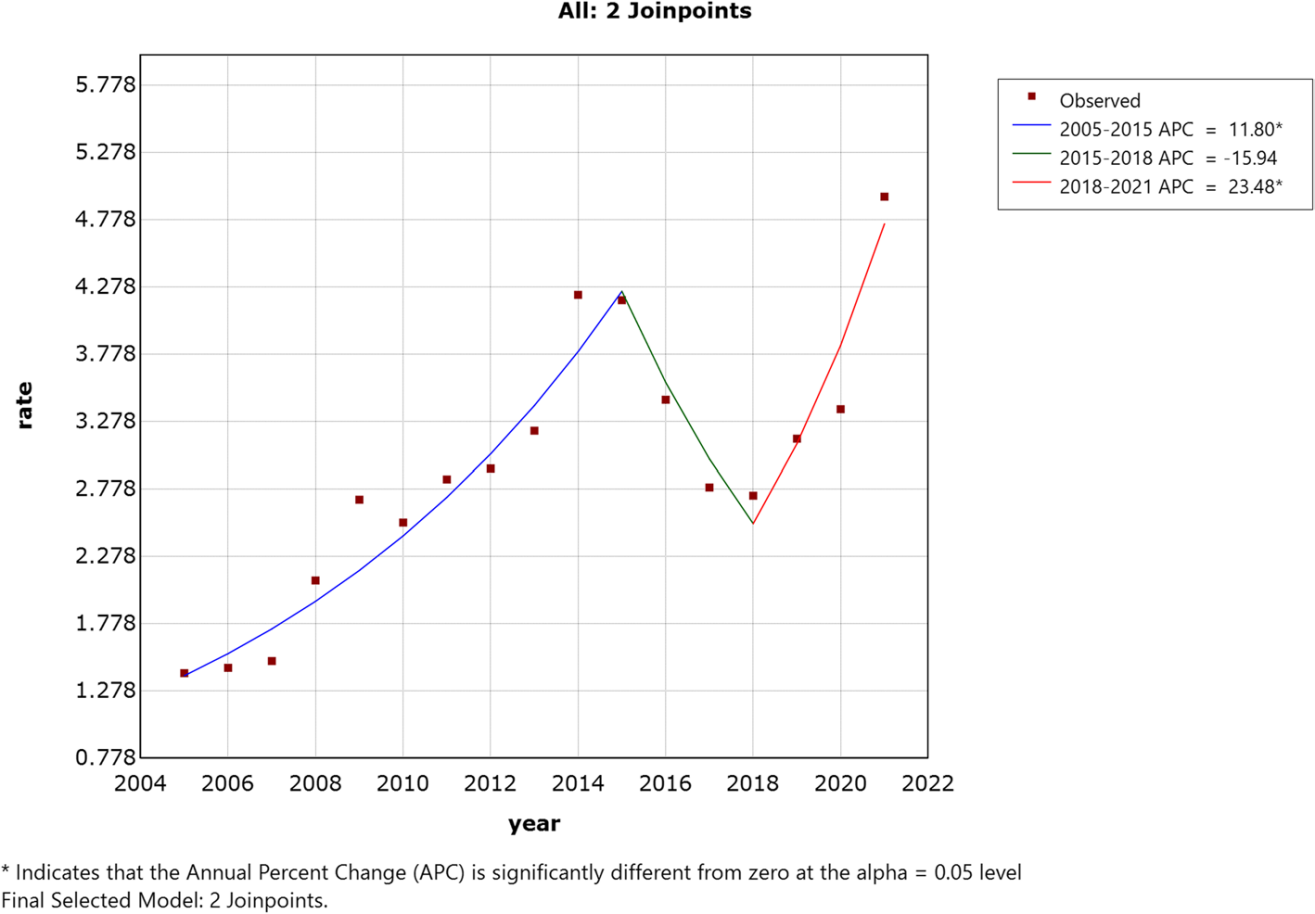
Join-point regression analysis of trends in the incidence of brucellosis in China, 2005–2021

The overall trend of the human brucellosis epidemic in China from 2005 to 2021 showed an upward trajectory, with the incidence rate increasing from 1.41/100,000 to 4.95/100,000. This change trend can be divided into three stages. The period from 2005 to 2014 exhibited a stable rising stage, with the incidence rate hovering around 2.49/100,000. The years 2015 to 2018 marked a declining stage, although the incidence remained higher than the national average in the 2005–2014 period. The years 2019 to 2021 constituted a rapid rise stage, with the epidemic experiencing a significant upsurge in 2019 and reaching its peak in 2021. Join-point regression analysis identified a total of two turning points, occurring in 2015 and 2018, as illustrated in Fig. 1.

The APC and AAPC results for human brucellosis incidence by region showed an overall upward trend from 2005 to 2021, with an average annual increase of 7.5–33.3%, with statistically significant differences in AAPC trends in all regions except Northeast and North China (P < 0.05). Among them, human brucellosis in South China experienced a process of deterioration followed by some improvement, with a rapid rise from 2005–2016 and a slow decline from 2016 to 2021. Central China was consistent with the change in the national trend of the incidence of human brucellosis. Eastern China and Northwest China showed a small upward trend from 2005 to 2009 and 2005 to 2011, respectively, followed by a significant increase in incidence trends through 2015, with Eastern China beginning to decline after 2015, while Northwest China rebounded and rose after declining from 2015 to 2018. Southwest China showed an upward trend since 2007, with an average annual rise of 23.7% (*P* < 0.05). The results were shown in Table 1.

**Table 1:** APC and AAPC for the incidence of brucellosis by region in China, 2005–2021

Region	Province	Year	APC(%)	AAPC(%)
Northeast China	Liaoning、Heilongjiang、Jilin	2005–2012	14.5*	4.4
		2012–2021	-2.8	
North China	Beijing、Tianjin、Hebei、Shanxi、Inner Mongolia	2005–2009	17.7*	5.2
		2009–2014	-1.2	
		2014–2017	-19.0	
		2017–2021	23.8*	
Eastern China	Shanghai、Zhejiang、Anhui、Fujian、Jiangxi、Shandong、Jiangsu	2005–2009	2.8	20.0*
		2009–2015	67.6*	
		2015–2021	-4.8	
South China	Guangdong、Guangxi、 Hainan	2005–2016	52.1*	33.3*
		2016–2021	-0.3	
Central China	Henan、Hubei、Hunan	2005–2015	46.1*	24.3*
		2015–2018	-32.6	
		2018–2021	33.4*	
Northwest China	Shaanxi、Qinghai、Ningxia、Xinjiang、Gansu	2005–2011	21.3*	20.2*
		2011–2015	64.9*	
		2015–2018	-25.2	
		2018–2021	24.4	
Southwest China	Chongqing、Sichuan、Guizhou、Yunnan、Tibet	2005–2007	-62.0	23.7*
		2007–2015	74.5*	
		2015–2021	16.0	

^⁎^*P* < 0.05

### Spatial changes in the national incidence of brucellosis from 2005 to 2021

**Fig. 2:**
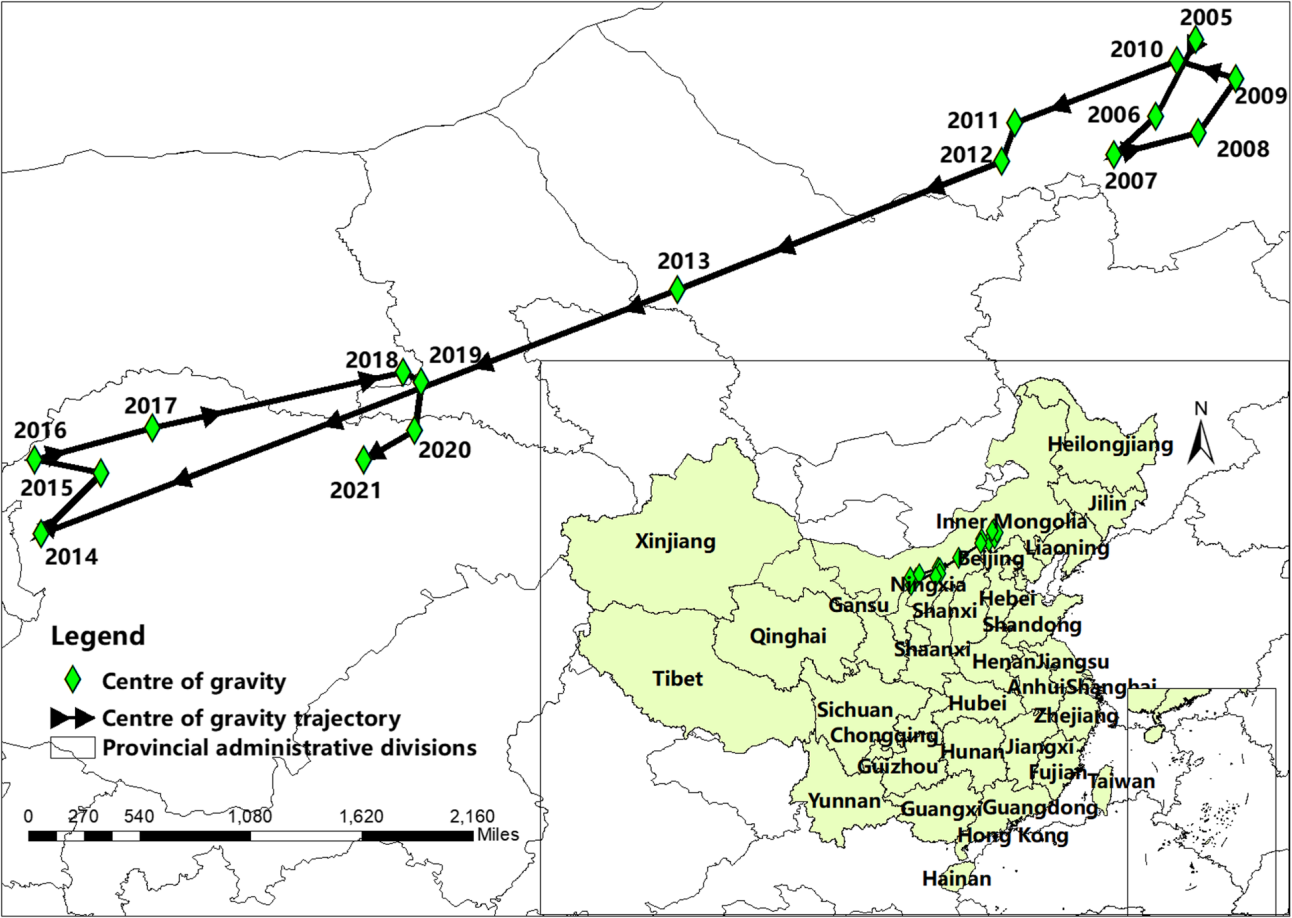
The center of gravity migration track of human brucellosis in China from 2005 to 2021

Between 2005 and 2021, the majority of human brucellosis cases were reported in northern China. The center of gravity for brucellosis shifted southwest from (115.5020°N, 43.2640°E) to (109.4590°N, 40.2092°E), covering a cumulative distance of 752.35 km. Initially concentrated in the central region of Inner Mongolia (2005–2010), there was a gradual southwest migration during 2010–2014. Despite remaining endemic in northern China from 2014–2018, post-2018 witnessed high outbreaks in the south. Figure 2 illustrates the trajectory of the annual migration.

The spatial distribution of human brucellosis incidence in China from 2005 to 2021 exhibited a relatively concentrated pattern, with regions of high incidence primarily concentrated in Inner Mongolia, Liaoning, Ningxia, Shandong, and Xinjiang. Notably, Inner Mongolia maintained the highest incidence rate for ten consecutive years. In Ningxia, the incidence rate was elevated in 2015 but steadily declined from 2016 to 2018, only to increase significantly in the subsequent three years. Furthermore, several southern provinces, such as Guangdong, Guangxi, Yunnan, Chongqing, and Sichuan, experienced a year-on-year increase in incidence rates. Conversely, lower incidence rates were concentrated in Jiangxi, Hunan, Shanghai, and Hainan.

The global autocorrelation analysis showed that the spatial autocorrelation Moran's *I* for the incidence of human brucellosis from 2005–2021 ranged from 0.145 to 0.398, all of which were positively correlated (*P* < 0.05), with significant aggregation. The results were shown in Table 2.

**Table 2:** Global autocorrelation Moran's *I* for the brucellosis epidemic in China, 2005–2021

Year	2005	2007	2009
Moran's *I*	0.145	0.200	0.180
Z-value	3.092	3.618	3.831
*P*-value	0.002	< 0.001	< 0.001
Year	2011	2013	2015
Moran's *I*	0.166	0.398	0.263
Z-value	3.641	4.391	2.931
*P*-value	< 0.001	< 0.001	0.003
Year	2017	2019	2021
Moran's *I*	0.291	0.295	0.329
Z-value	3.212	3.867	4.071
*P*-value	0.001	< 0.001	< 0.001

### Results of model fit comparison

The results were shown in Table 3. The R^2^ and Adjusted R^2^ values of the MGWR model were higher than those of the OLS and GWR models, at 0.838, 0.642 and 0.636, respectively. The AICc values of the MGWR model were smaller than those of the OLS and GWR models, at 639.626, 974.732 and 3493.899 respectively, suggesting that the MGWR model could better explain the effects of independent variables on the incidence of human brucellosis and should be better able to explain data with spatial and temporal characteristics.

**Table 3:** Comparison results of OLS, GWR and MGWR models

Models	AICc	*R*^*2*^	Adjusted R^2^
OLS	3493.899	0.642	0.633
GWR	974.732	0.636	0.629
MGWR	639.626	0.838	0.822

The mean, standard deviation, minimum, median and maximum values are used to describe the fit coefficients of the MGWR model, respectively, to describe the spatiotemporal characteristics of the disease. The results were shown in Table 4.

**Table 4:** Descriptive statistics of the standardized regression coefficients for each explanatory variable of the MGWR model

	**Mean ± SD**	**Min**	**Med**	**Max**	***P*-value**
AAT(℃)	-0.473 ± 0.004	-0.485	-0.473	-0.467	< 0.001
ARH(%)	-0.054 ± 0.043	-0.177	-0.040	-0.006	0.744
AP(mm)	-0.053 ± 0.070	-0.199	-0.028	0.020	0.067
ASH(hours)	0.131 ± 0.016	0.090	0.137	0.154	0.105
NOCS(million)	0.103 ± 0.003	0.095	0.103	0.108	0.964
NOSS(million)	-0.407 ± 0.309	-0.747	-0.523	0.377	0.007
BP (million tons)	-0.388 ± 0.322	-1.020	-0.408	0.425	< 0.001
MP (million tons)	0.859 ± 0.179	0.294	0.896	1.025	< 0.001
DP (million tons)	0.616 ± 1.018	-0.095	0.143	3.558	< 0.001
GRP(billion yuan)	-0.014 ± 0.003	-0.022	-0.013	-0.007	0.009
GPP(billion yuan)	0.033 ± 0.005	0.030	0.031	0.051	0.658
HE(billion yuan)	-0.055 ± 0.085	-0.465	-0.029	0.170	0.015

*AAT* Average annual temperature, *ARH* Average relative humidity, *AP* Annual precipitation, *ASH* Annual sunshine hours, *NOCS* Number of cattle stock, *NOSS* Number of sheep stock, *BP* Beef production, *MP* Mutton production, *DP* Dairy production, *GRP* Gross regional product, *GPP* Gross pastoral product, *HE* Health expenditure

### Spatial and temporal distribution of regression coefficients in the MGWR

**Fig. 3:**
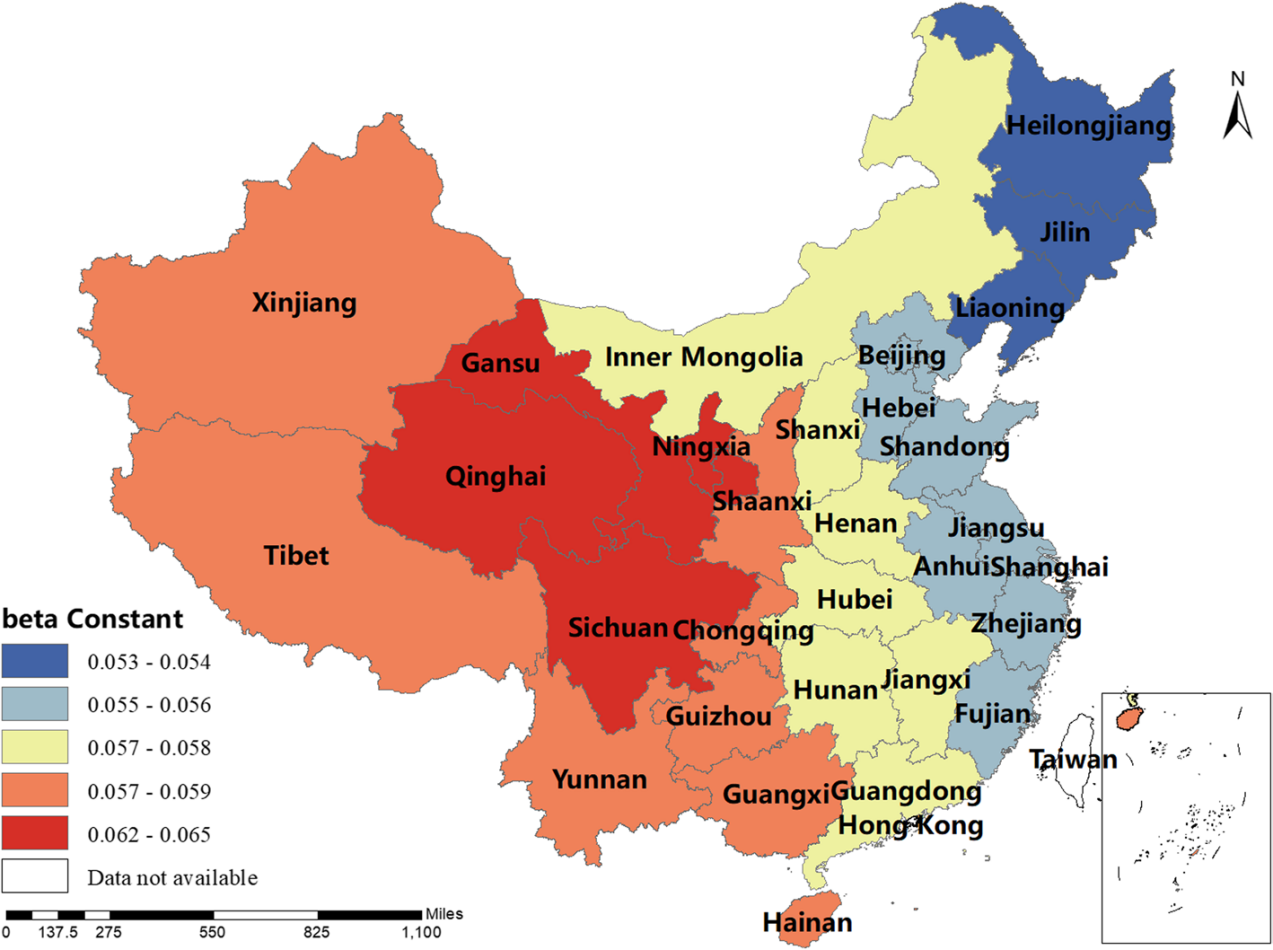
Spatial and temporal distribution of regression coefficients for Constant

**Fig. 4:**
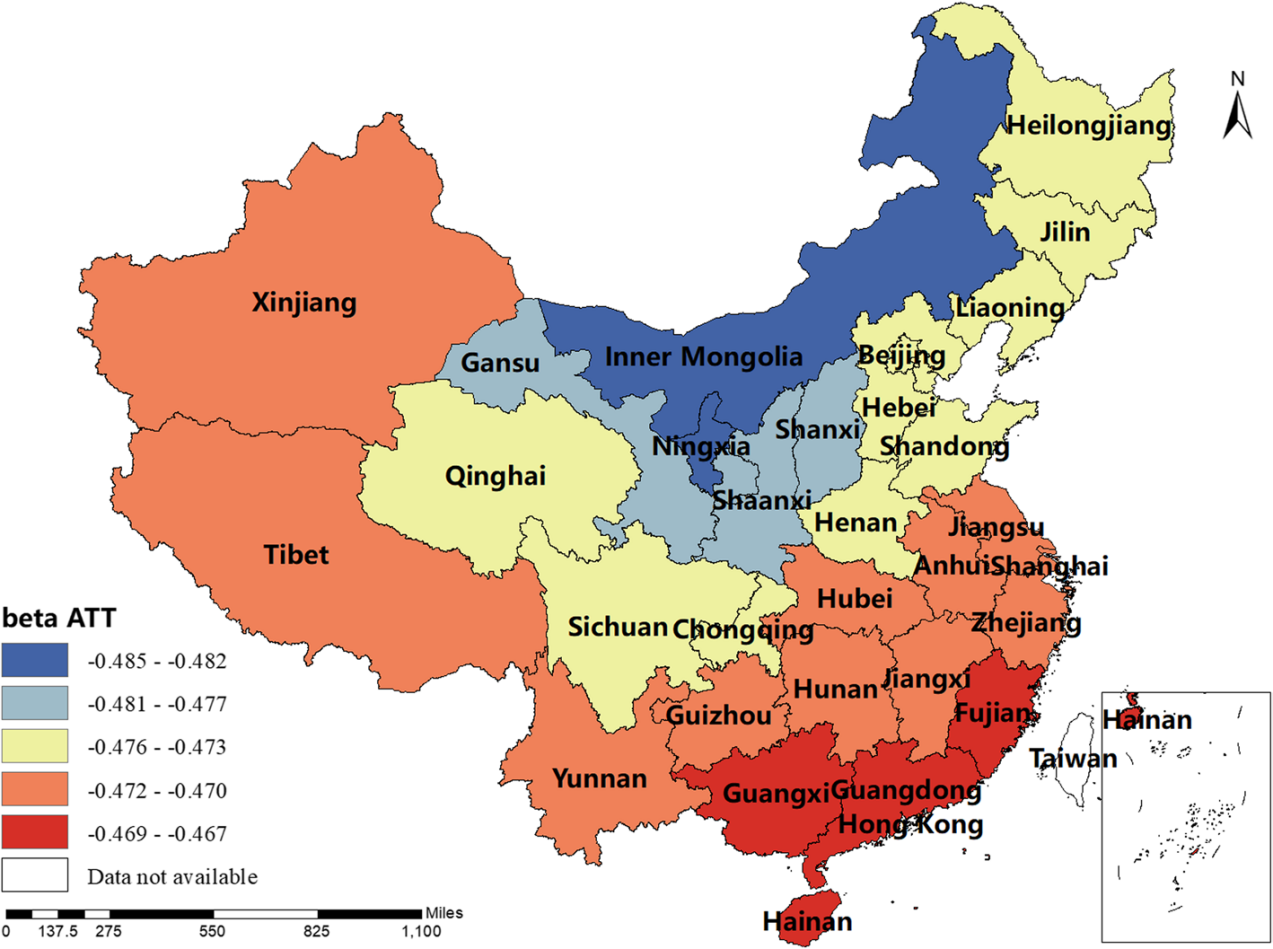
Spatial and temporal distribution of regression coefficients for AAT

**Fig. 5:**
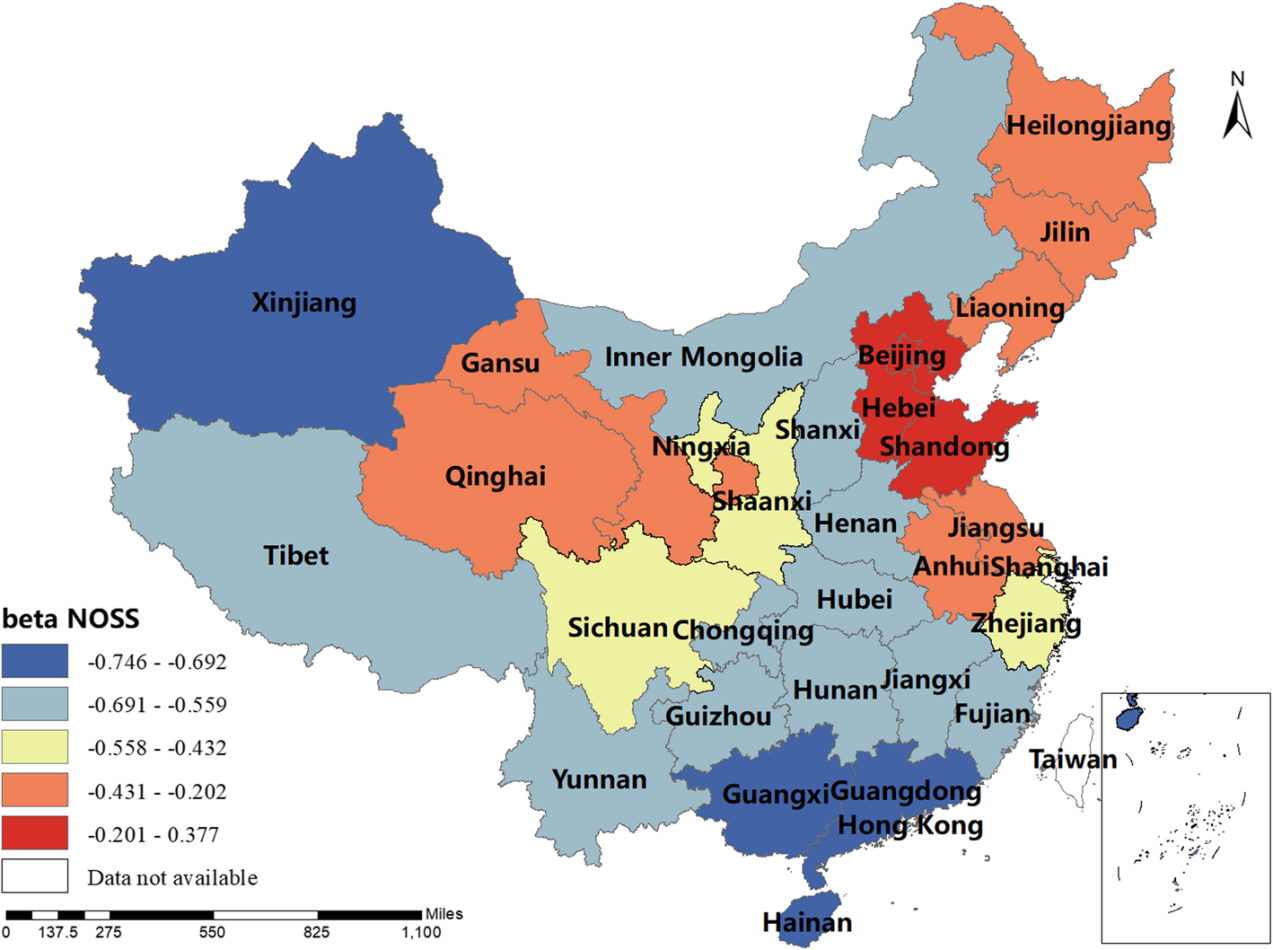
Spatial and temporal distribution of regression coefficients for NOSS

**Fig. 6:**
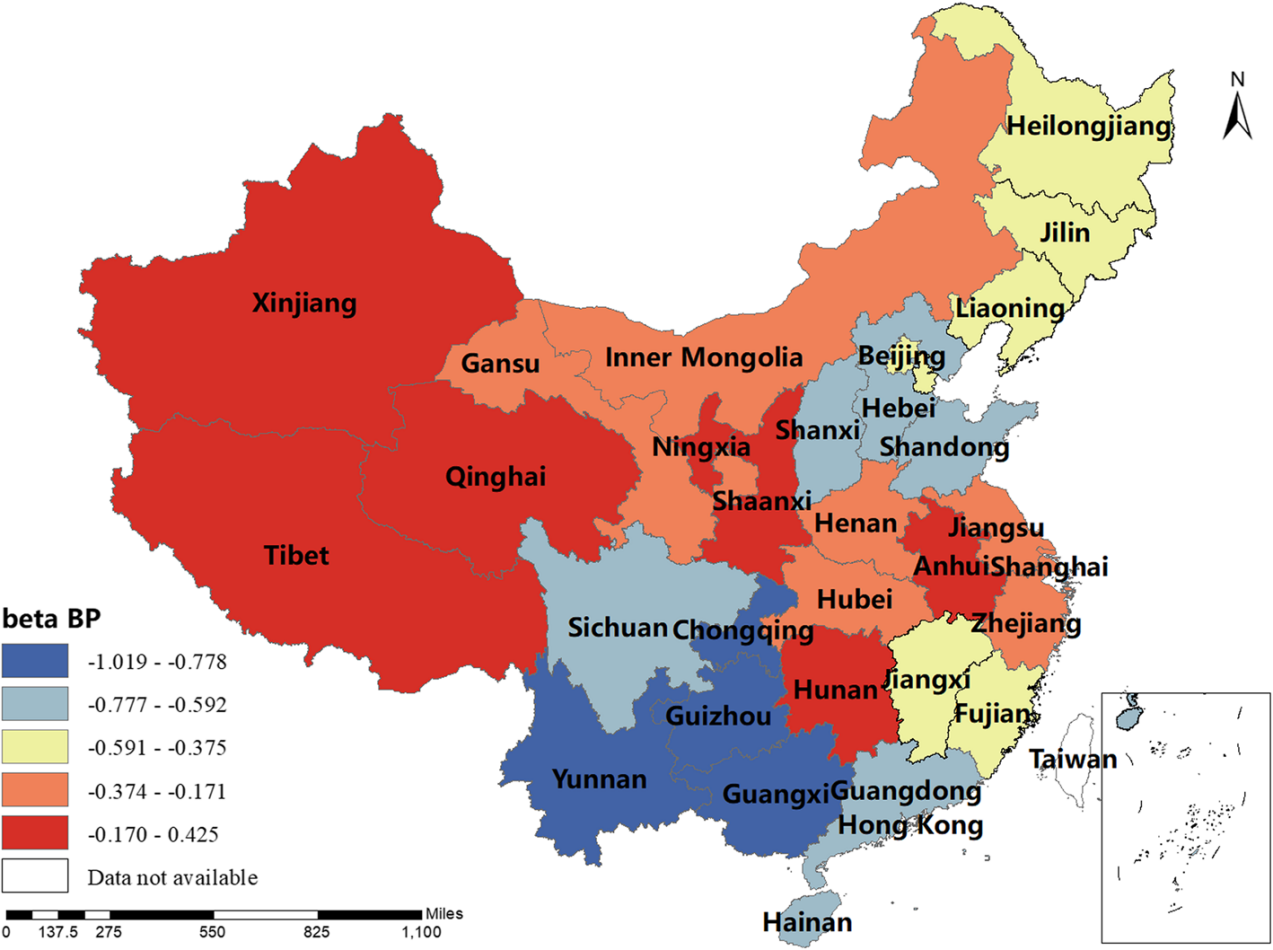
Spatial and temporal distribution of regression coefficients for BP

**Fig. 7:**
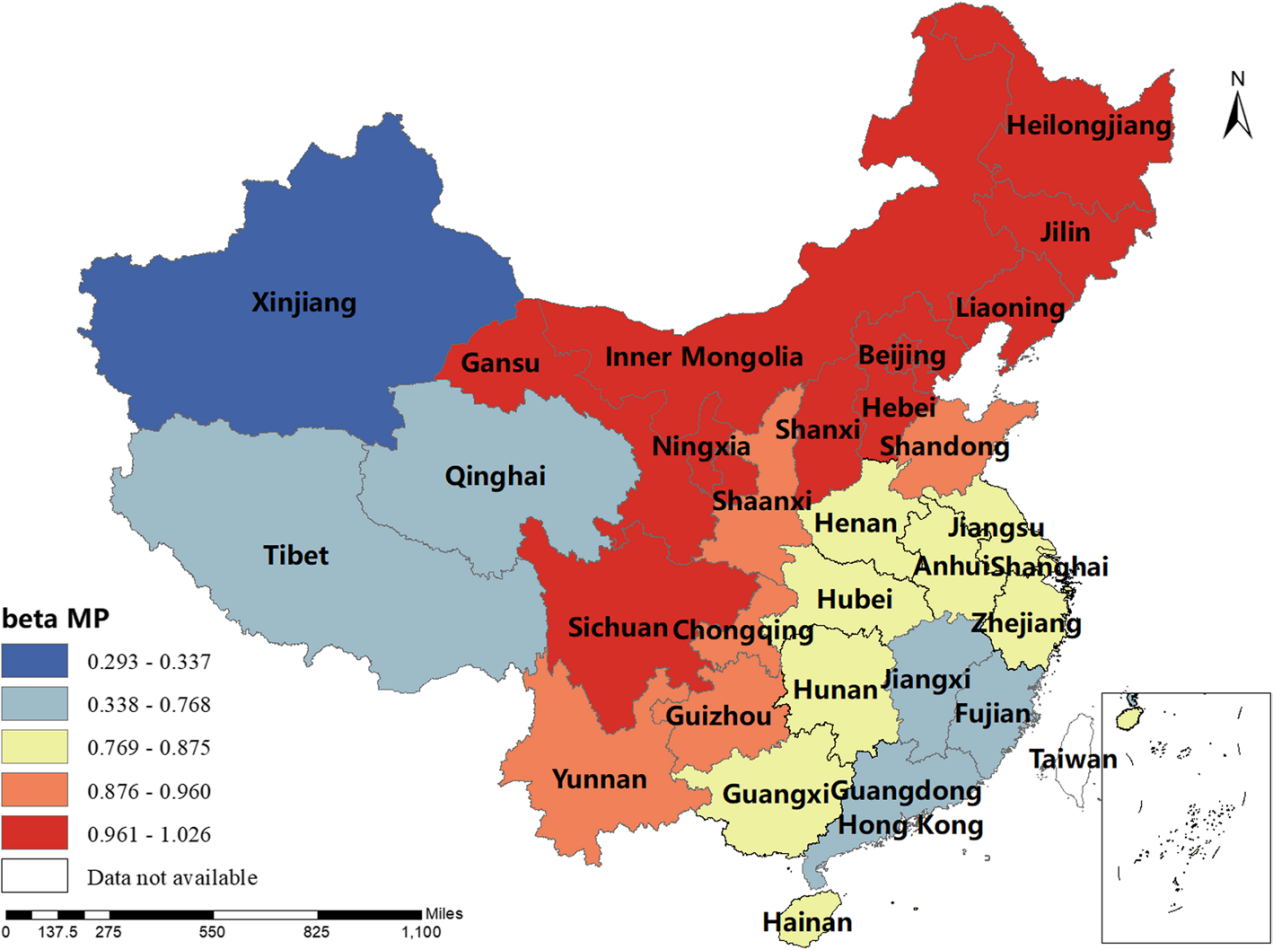
Spatial and temporal distribution of regression coefficients for MP

**Fig. 8:**
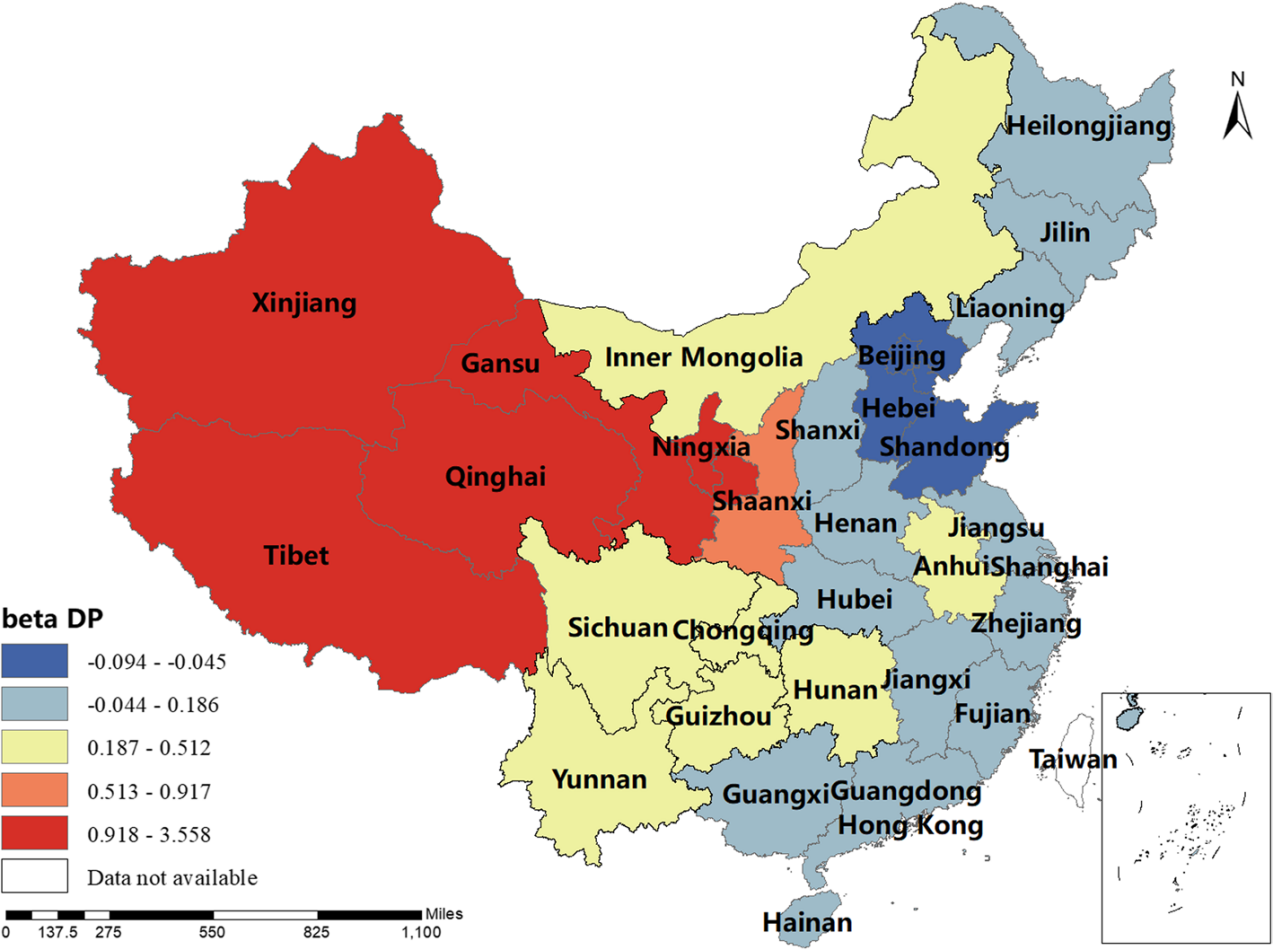
Spatial and temporal distribution of regression coefficients for DP

**Fig. 9:**
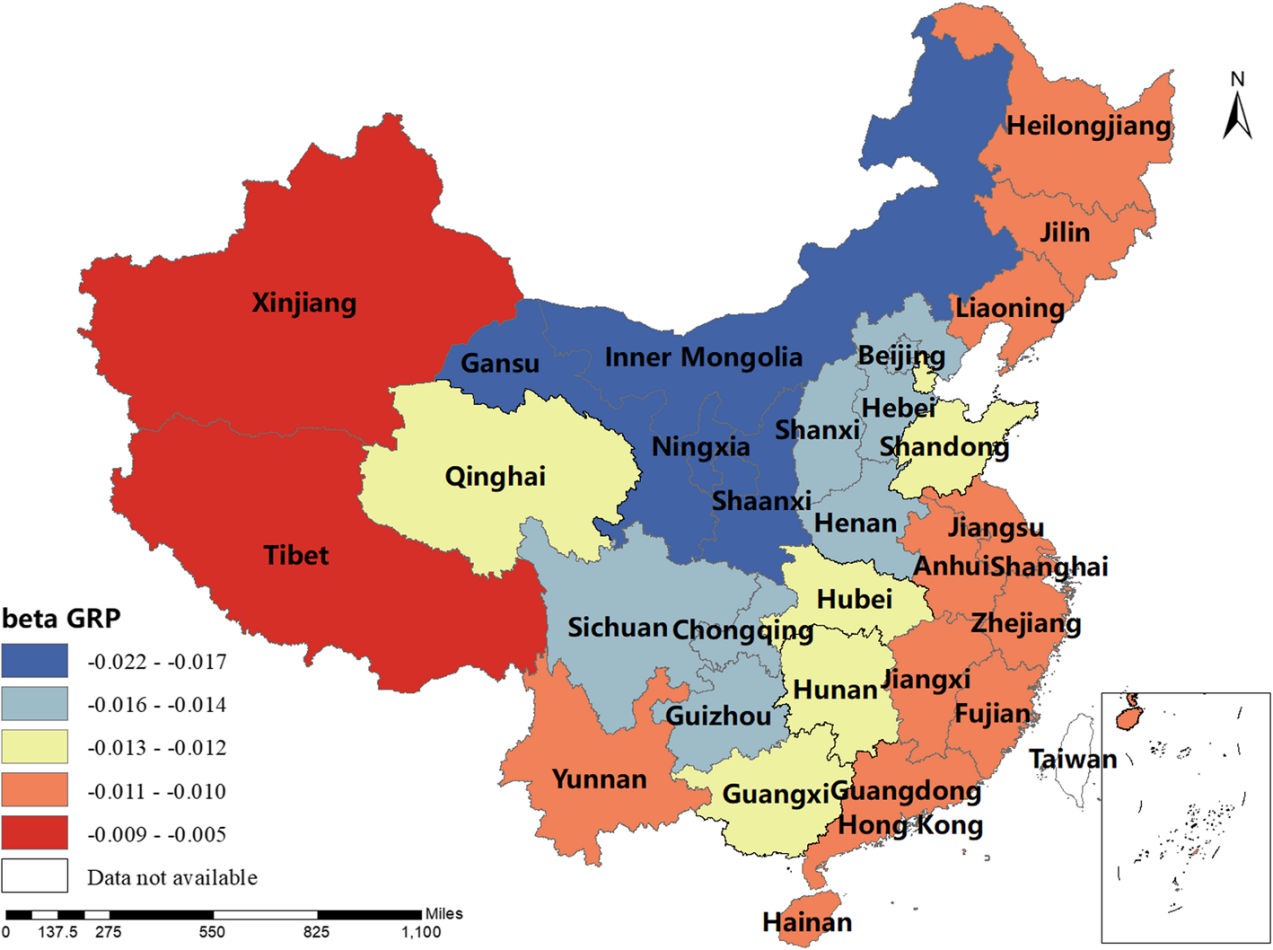
Spatial and temporal distribution of regression coefficients for GRP

**Fig. 10:**
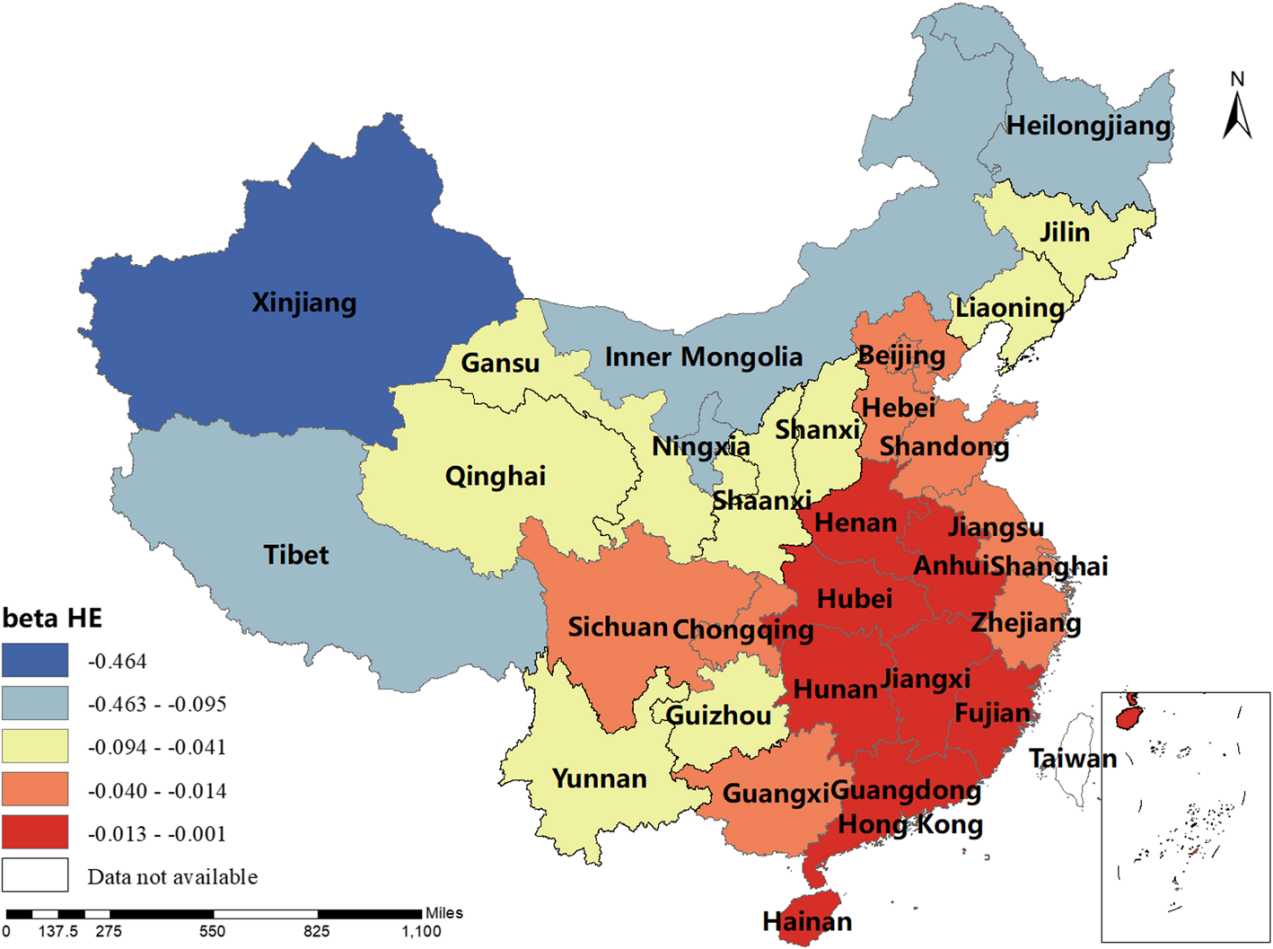
Spatial and temporal distribution of regression coefficients for HE

The magnitude and sign of the MGWR model parameter estimates reflect the degree and direction of influence of each factor of interest on the incidence of human brucellosis in different regions. Only variables that are statistically different were shown here. The spatial distribution of the constant term reflected the spatial variation in the "baseline level" of the incidence of the disease at a value of zero for each of the influencing variables, i.e. the influence of factors other than those considered in this study.The spatial distribution of the estimated AAT coefficients showed that most regions have negative coefficient estimates, indicating that the lower the temperature, the higher the incidence of human brucellosis. This factor played a stronger role in Northern China. The majority of regions had negative NOSS coefficient estimates, indicating that the higher the number of sheep stock, the lower the incidence of human brucellosis. This factor had a stronger effect in Guangdong, Guangxi and Xinjiang. The estimated value of the coefficient of BP was positive in Shaanxi, Hunan, Tibet, Qinghai and Xinjiang, i.e. the higher the beef production, the higher the incidence of human brucellosis. The coefficient estimates of MP were all positive, indicating that mutton production was positively correlated with the incidence of human brucellosis. This factor plays a significant role in Inner Mongolia, Heilongjiang, Jilin, Liaoning, Gansu, Ningxia and Sichuan. The coefficient estimates for DP were positive in most regions, i.e. the higher the dairy production, the higher the incidence of human brucellosis. The Northwest China and Tibet were more affected by this factor.The coefficient estimates for GRP were all negative, indicating that the higher the regional GDP, the lower the incidence of human brucellosis. This factor was more obvious in Xinjiang and Tibet.The coefficient estimates of HE were all negative, i.e. health expenditure was negatively correlated with the incidence of human brucellosis. Central and Southern China were more strongly influenced by this factor. The results were shown in Figs. 3, 4, 5, 6, 7, 8, 9, 10.

## Discussion

Currently, research on brucellosis predominantly focuses on descriptive epidemiology, including diagnosis, treatment, and immunization. However, brucellosis, being a zoonotic disease, is influenced by numerous factors, such as the geographical and natural environment, the production and lifestyle of the inhabitants, and the number, distribution, and movement of infectious livestock [16, 17]. Furthermore, both the natural and social environments vary across regions in China. Therefore, it becomes even more critical to leverage local favorable conditions and explore precise strategies for the prevention and treatment of human brucellosis.

The results of the Join-point model, constructed using human brucellosis data from 2005 to 2021, indicate that the overall incidence of human brucellosis in China exhibited an upward trend. Although the number of cases began to decline after 2015, the incidence remained high. The factors contributing to the rebound and increase after 2018 are multifaceted and likely related to several factors, including the development of the livestock economy, historical infection hotspots, the movement of infectious sources, and varying regional priorities in addressing human brucellosis.

Furthermore, this study found that the AAPC of human brucellosis incidence was positive in all regions, with a more significant annual increase observed in South, Central, and Southwest China. This finding aligns with previous research, which identified a tendency for brucellosis to spread to southern provinces [18, 19]. The rise in human brucellosis incidence in southern provinces may be attributed to factors such as increased travel and heightened demand for lamb and beef. Consequently, it is crucial to strengthen agricultural market management and supervision, both domestically and internationally, to prevent contaminated meat and dairy products from entering the market. Additionally, the increase in the south may be due to historical lower endemicity in the region, leading to underdeveloped countermeasures, insufficient quarantine immunity, irregular slaughtering practices, and inadequate treatment of infectious sources, ultimately intensifying the disease's epidemic. Thus, it is imperative to enhance the management, training, and health education efforts of relevant departments and occupational groups in the southern region [20–23].

In this study, the national share of brucellosis in Northwest China surged from 4.41% in 2005 to 23.66% in 2021. Similarly, the national share of brucellosis in the southwest region increased from 0.11% in 2005 to 1.58% in 2021, consistent with the findings of Tao et al. [24]. The westward shift may be attributed, in part, to a decrease in cases in the northeastern region, contributing to a decline in the country's overall share. Concurrently, the development of the livestock industry in the west has increased the likelihood of relevant practitioners coming into contact with infected animals.

A brief shift of the centre of gravity of morbidity back towards the north in 2016–2018 was found on the migration map.Consideration of possible reasons for the increase in incidence in Inner Mongolia and subsequent rebound in neighbouring provinces. Firstly, market prices for beef and lamb have been on the rise since 2016, attracting more individuals involved in farming, buying, selling, and trading. Secondly, the movement of livestock has increased in tandem with demand, leading to the spread of brucellosis through diseased animals due to a lack of proper quarantine and inspection. For instance, genotypic studies have indicated that brucellosis strains in Inner Mongolia are related to strains from neighbouring provinces [25]. Thirdly, resources invested in brucellosis control may have decreased, with activities based on the One Health principle declining after successive years of decline—an impression gleaned from field visits to endemic areas.

We also observed a significant positive spatial correlation in the incidence of human brucellosis across all regions of China from 2005 to 2021. This finding indicates a spatial clustering phenomenon in the distribution of human brucellosis patients, suggesting the presence of spatial non-stationarity. This spatial non-stationarity can be attributed, to some extent, to spatial heterogeneity in the influence of related factors across different regions. Therefore, we further constructed the MGWR model for human brucellosis incidence and related factors to quantify the spatial variability in the influence of these factors on human brucellosis incidence.

In our study, we identified a negative effect of average annual temperature on the incidence of human brucellosis, consistent with results from studies in Iran and Hebei Province, China [26, 27]. This negative effect may be attributed to lower temperatures and higher humidity, which create favorable conditions for the long-term survival of Brucella spp. in the environment. During colder weather, livestock tend to be housed indoors, leading to prolonged human-livestock contact and an increased risk of infection. Meteorological factors had a more substantial impact on the incidence of brucellosis in northern regions, suggesting the need for key monitoring during temperature fluctuations and the promotion of proper livestock breeding practices and disinfection of breeding environments.Furthermore, human brucellosis incidence was positively correlated with beef, mutton, and dairy production but negatively correlated with the number of sheep stock. This association is linked to the fact that the digestive tract is a primary transmission route for human brucellosis. In recent years, the rise in demand for beef, mutton, and dairy products, along with inadequate circulation, storage, and handling of these food products, has led to frequent cases of foodborne human brucellosis infection [28]. Notably, the occurrence of human brucellosis in Xinjiang, Tibet, Ningxia, and Gansu was strongly associated with beef and dairy production, highlighting the importance of enhancing food safety management in these areas. Conversely, mutton production had a more substantial impact on human brucellosis incidence in regions like Inner Mongolia, Heilongjiang, Beijing, Tianjin, and Sichuan. The varying influence of different food production types on human brucellosis incidence may be related to different dietary habits, suggesting the need for targeted health education based on local dietary characteristics.

The year-end number of sheep stock reflects the overall health status of sheep during that period. A higher number at year-end corresponds to better sheep health and a lower human brucellosis infection rate. Herders tend to become infected through direct contact with brucella-infected sheep or their products [29]. Therefore, human brucellosis is more likely to occur in northern and northwestern regions with larger sheep populations. We strongly recommend implementing control measures in these areas with a history of sheep or goat exposure.The national program advocates for a mass animal vaccination strategy in high-endemic areas and the implementation of a "quarantine-vaccination-slaughter" policy (i.e., vaccinating negative individuals based on quarantine results and safely slaughtering animals that test positive) in less-affected areas. Success has been achieved in some areas, with Xinjiang being a notable example. As one of the endemic regions in China, Xinjiang has conducted a large-scale animal brucellosis vaccination campaign since 2016 [30], resulting in a significant decline in the incidence of human brucellosis. A study in the Hami region of Xinjiang demonstrated a noteworthy decrease in local brucellosis infection rates in cattle and sheep from 2017 to 2019.Lastly, areas with higher gross regional development and health expenditure exhibited lower human brucellosis incidence, underscoring the importance of economic support for the effective implementation of strategies and measures to control and eradicate brucellosis [31–33].

In addressing human brucellosis in China, tailored prevention and control measures should align with local conditions. Southern regions like Guangdong and Guangxi can collaborate with meteorological departments for real-time temperature monitoring, predicting key disease prevention periods. In Beijing and Hebei, overseeing livestock health and practitioners will be crucial, along with researching local immunization effectiveness. Northwestern areas such as Tibet and Hunan should enhance food safety management for beef and dairy, expanding animal product market quarantine. Meanwhile, in economically diverse regions like Hubei and Jiangxi, increasing health budgets will support local brucellosis prevention and control efforts.

The spatial distribution map of the parameter estimates revealed significant spatial differences in the coefficient estimates of the factors of interest across each region. This finding underscores the need to develop regionalized plans and strategies for the prevention and control of brucellosis, taking into account the spatial characteristics of these factors and their local relationships with brucellosis incidence.

Additionally, our study observed both positive and negative signs in the coefficient estimates of the MGWR model, indicating that the MGWR method better reflects spatial non-stationarity compared to the OLS method. Model evaluation results also indicated that MGWR outperforms the traditional GWR model, providing more reliable parameter estimation results. These findings emphasize the importance of considering differential scales of action and spatial heterogeneity to enhance model accuracy.

Our study focused on the spatial–temporal evolution of human brucellosis in China, providing valuable insights into the dynamics of brucellosis transmission. We also revealed the spatial–temporal heterogeneity of human brucellosis influencing factors at multiple spatial scales, spanning from meteorological to socioeconomic factors. This may aid in the development of more precise prevention and control strategies. However, certain multidimensional epidemiological factors, such as high-risk population factors, were not included due to limitations in data acquisition. Therefore, we plan to continue expanding our dataset to enhance the applicability and robustness of the model.

## Conclusion

The eradication of brucellosis is a systematic and complex process aimed at controlling and eventually eliminating the disease at its source, conducted in stages. Efforts should focus on developing effective prevention and control strategies that consider regional differences, including preventing contact transmission, addressing food-borne transmission, strengthening laboratory safety measures, enforcing strict import controls, providing economic support for active surveillance, and implementing vaccination programs and selective culling.

Moreover, there is a need for further research in health economics evaluation and related studies to promote cross-sectoral cooperation in brucellosis control. Enhancing information exchange on brucellosis control between regions is crucial. Ultimately, the goal is to bring the incidence of brucellosis within a manageable range and work towards its eventual eradication.

## Data Availability

The datasets analyzed during the current study are available from the corresponding author on reasonable request. The URL link of the dataset is: https://www.phsciencedata.cn/Share/en/data.jsp?id=8e3f97d5-bbac-4fc1-9e93-d2557dd01ffc&show=0.

## Abbreviations

OLS: Ordinary least squares
GWR: Geographically weighted regression
MGWR: Multiscale geographically weighted regression
APC: Annual percent change
AAPC: Average annual percent change
